# Variation in communication of side effects in prostate cancer treatment consultations

**DOI:** 10.1038/s41391-024-00806-2

**Published:** 2024-02-23

**Authors:** Timothy J. Daskivich, Aurash Naser-Tavakolian, Rebecca Gale, Michael Luu, Nadine Friedrich, Abhi Venkataramana, Dmitry Khodyakov, Edwin Posadas, Howard Sandler, Brennan Spiegel, Stephen J. Freedland

**Affiliations:** 1 Department of Urology, Cedars-Sinai Medical Center, Los Angeles, CA USA; 2 Cedars-Sinai Center for Outcomes Research and Education (CS-CORE), Cedars-Sinai Medical Center, Los Angeles, CA USA; 3 Department of Biostatistics, Cedars-Sinai Medical Center, Los Angeles, CA USA; 4 Institute of Urology, University of Southern California, Los Angeles, CA USA; 5 RAND Institute, Santa Monica, CA USA; 6 Department of Medicine, Division of Medical Oncology, Cedars-Sinai Medical Center, Los Angeles, CA USA; 7 Department of Radiation Oncology, Cedars-Sinai Medical Center, Los Angeles, CA USA; 8 Department of Medicine, Divisions of Gastroenterology and Health Services Research, Cedars-Sinai Medical Center, Los Angeles, CA USA; 9 Section of Urology, Durham VA Medical Center, Durham, NC USA

**Keywords:** Medical research, Cancer therapy

## Abstract

**Background:**

Effective communication of treatment side effects (SE) is critical for shared decision-making (SDM) in localized prostate cancer. We sought to qualitatively characterize how physicians communicate SE in consultations.

**Methods:**

We transcribed 50 initial prostate cancer treatment consultations across nine multidisciplinary providers (Urologists, Radiation Oncologists, Medical Oncologists) at our tertiary referral, academic center. Coders identified quotes describing SE and used an inductive approach to establish a hierarchy for granularity of communication: (1) not mentioned, (2) name only, (3) generalization(“high”), (4) average incidence without timepoint, (5) average incidence with timepoint, and (6) precision estimate. We reported the most granular mode of communication for each SE throughout the consultation overall and across specialty and tumor risk.

**Results:**

Among consultations discussing surgery (*n* = 40), erectile dysfunction (ED) and urinary incontinence (UI) were omitted in 15% and 12%, not quantified (name only or generalization) in 47% and 30%, and noted as average incidence without timeline in 8% and 8%, respectively. In only 30% and 49% were ED and UI quantified with timeline (average incidence with timeline or precision estimate), respectively. Among consultations discussing radiation (*n* = 36), irritative urinary symptoms, ED, and other post-radiotherapy SE were omitted in 22%, 42%, and 64–67%, not quantified in 61%, 33%, and 23–28%, and noted as average incidence without timeline in 8%, 22%, and 6–8%, respectively. In only 3–8% were post-radiotherapy SE quantified with timeline. Specialty concordance (but not tumor risk) was associated with higher granularity of communication, though physicians frequently failed to quantify specialty-concordant SE.

**Conclusions:**

SE was often omitted, not quantified, and/or lacked a timeline in treatment consultations in our sample. Physicians should articulate, quantify, and assign a timeline for SE to optimize SDM.

## Introduction

Shared decision making (SDM)—the guidelines-endorsed standard of care for counseling men with prostate cancer [1]—involves education of patients regarding risks and rewards of therapy and reaching a collaborative treatment decision based on patient preferences for balancing these tradeoffs [2, 3]. Key to this process is clear communication of treatment-related side effects (SE). Both describing and quantifying major SE are critical for the patient to weigh the potential risks of treatment against its rewards (namely, reduction in risk of cancer progression and mortality) [4]. Since patients often have little insight into these tradeoffs [5], it is challenging for the physician to educate the patient in a brief period of time to ensure informed SDM.

Despite the key role of adequate communication of SE in SDM, little is known about how SE are communicated in practice. We previously analyzed variation in communication of the survival benefit related to treatment—the “rewards” side of treatment tradeoffs—during treatment consultations and found substantial variation in the quality of risk communication [6]. In 40 consultations, physicians often failed to communicate the reduction in cancer mortality associated with treatment; cancer mortality was reported without treatment in 38%, with treatment in 10%, and in only 29% of consultations was cancer mortality reported both with and without treatment [6]. To our knowledge, there has been no similar analysis of how treatment-related SE are communicated by counseling physicians.

In this study, we conducted a qualitative analysis of initial treatment consultations of men with newly diagnosed prostate cancer at our institution across 9 multidisciplinary providers who typically counsel these men. We sought to qualitatively characterize how physicians communicate SE and to establish a framework for quality of risk communication. We then sought to better understand how tumor risk and physician specialty affect quality of risk communication regarding SE. We hypothesized that wide variation would exist in whether individual SE were discussed or quantified during the consultation and that this variation would persist in subgroups of specialty and tumor risk. By categorizing how physicians communicate these risks, we aimed to characterize the varying informational quality in reporting risk of SE and gain insight into best practices to optimize SDM.

## Materials and methods

### Study cohort

We recruited men with newly diagnosed Gleason ≤ 7, clinical stage ≤ T2c prostate cancer undergoing initial outpatient consultations among the practices of three urologists, three medical oncologists, and three radiation oncologists within our institution, a tertiary referral center. We excluded patients <18 years and non-English speakers. The study was IRB approved (Pro#00053972).

### Informed consent

Subjects and physicians were informed in the written consent that the study would assess “communication of risks” since more specific disclosure may have influenced content discussed. At the study conclusion, we debriefed subjects about the specific hypothesis. This IRB-approved approach was justified under 45CFR46.116(d).

### Consultation coding

Outpatient consultations were digitally recorded and transcribed verbatim. Quotes related to treatment-related SE were extracted from transcripts. Four coders independently analyzed these quotes using an inductive coding approach to characterize SE type, mode of communication, and whether a timeline was mentioned. Our coding approach was informed by our expertise in prostate cancer treatment and our previous work, in which we characterized patient preferences [7] and physician variation in communication [6] of competing risks of mortality.

Coders then met to confirm a final codebook and establish a hierarchy for increasing granularity of communication: (1) not mentioned (2), name only (without risk quantification) (2), generalization (“high”) (3), average percent incidence without timepoint (4), average percent incidence with timepoint (5), precision estimate accounting for patient-level characteristics. Coders then retrospectively applied this hierarchy to the entire dataset.

### Statistical analysis

**Fig. 1:**
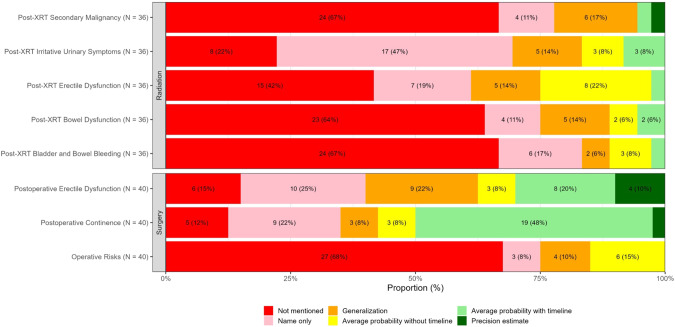
Variation in mode of communication of side effects by treatment type.

The most granular mode of communication used to describe each SE throughout each consultation was described using counts/proportions. Comparisons by specialty and tumor risk were performed using the Chi-square test via Monte-Carlo simulation with 2000 replicates [8], with post-hoc Tukey’s test for pairwise comparisons, and adjustment for multiple comparisons using the Holm correction. For comparisons by specialty and tumor risk, the denominator was the total number of opportunities to discuss treatment-specific SE (e.g., for each surgical consultation, we compared communication for each surgical SE described in Fig. 1). All statistical analyses were performed using R statistical software (version 4.2.0; R Foundation, Vienna, Austria) with two-sided tests and a significance level of 0.05.

## Results

The study cohort included 50 men with demographics reflective of a typical US prostate cancer population (Appendix Table 1).

### Surgical SE

Surgery was discussed in 40 of 50 consultations (80%). Coders identified three discrete surgery-related SE discussed in these consultations: postoperative erectile dysfunction (ED), postoperative urinary incontinence (UI), and operative risks. Operative risks included complications in the perioperative setting and during convalescence (e.g., pain, surgical site infection, lymphocele, urine leak).

There was wide variation in the most detailed form of communication used at any point during the consultation to describe major surgical SE (Fig. 1, Appendix Table 2). For example, risk of postoperative ED was not mentioned in 15%(6/40) of consultations, mentioned in name only in 25%(10/40), generalized (e.g., “high”) in 22%(9/40), noted as an average probability with and without a timepoint in 20%(8/40) and 8%(3/40), respectively, and as a precision estimate accounting for patient-level characteristics in 10%(4/40). Similarly, risk of UI was not mentioned in 12%(5/40) of consultations, mentioned in name only in 22%(9/40), generalized in 8%(3/40), noted as an average probability with and without a timepoint in 48%(19/40) and 8%(3/40), respectively, and as a precision estimate in 2%(1/40). Operative risks were frequently omitted (68%(27/40)) or not quantified (name only, 8%(3/40), generalization, 10%(4/40)).

These modes of communication encompassed a wide range of informational quality (illustrative quotes in Table 1). When noted in name only or as a generalization, there was no numeric quantification of risk: For example, “patients do well after surgery, with minimal effect on potency.” When described as an average incidence without a timepoint, risk was articulated as an average population risk without a discrete timepoint for that risk, as in “about 50% will have some form of ED.” When described as an average incidence with a timepoint, the average risk was assigned a clear timepoint for risk, as in “less than 10% of men need a pad beyond a year.” When described as a precision estimate, risk was quantified at a timepoint and personalized based on the characteristics of the individual: “So you are super young and your [baseline] erectile function is excellent…I would say your likelihood of regaining your baseline function in a year for both continence and erectile function is upwards of like 90%.” While accuracy of numeric estimates is difficult to assess retrospectively due the many factors affecting outcomes, the numeric values when reported were heterogeneous in composition (i.e., risk of experiencing/avoiding side effect, timeline) and range (Appendix Table 3).

**Table 1:** Modes of communication of major postoperative and post-radiation side effects.

Mode of Communication	Frequency (*n*, %)	Illustrative Quote
Postoperative Erectile Dysfunction		
Not mentioned	6 (15%)	
Name Only	10 (25%)	“The other thing that patients notice after surgery is issues with erectile function.”
Generalization	9 (22%)	“Sexual function, if you’re coming into it with some issues the likelihood that at 74 you would regain your sexual function from baseline after surgery is pretty low.”
Average Probability without Timeline	3 (8%)	“It’s about 33–50% of men will have some form of erectile dysfunction and that can have a various range so not ever guy when I say erectile dysfunction would be flaccid it would mean that some guys would be softer, notice it’s harder to get an erection, need things like Viagra or Cialis to have an erection.”
Average Probability with Timepoint	8 (20%)	“The erectile dysfunction is also 100% initially and this takes 12–24 months to get better. It gets better in most patients but in 20%, it doesn’t.”
Precision Estimate	4 (10%)	“It is just the erectile function of your penis that can be affected. And the best predictor of how well you’re doing to do in terms of your erectile function after surgery is what you bring to the table beforehand based on your age and based on your erectile function. So you are super-young and your erectile function is excellent… I would say that your likelihood of regaining your baseline function in a year for both continence and erectile function is upwards of like 90%.” “So, it takes about a year to go back to normal [potency]. Now, if you have good erections now -- you’re sixty-two -- the likelihood that you are going to regain your baseline potency is—is pretty high. So, being sixty-two and having perfect erections now, I’d say you’re eighty percent likelihood of getting to that at a year…so my guess is that you’re going to have an earlier recovery of both of these functions than patients who are older.
Postoperative Urinary Incontinence		
Not mentioned	5 (12%)	
Name Only	9 (22%)	“…the broad risks are urinary leakage or incontinence…”
Generalization	3 (8%)	“Patients do really well after the surgery functionally, [with] minimal effect on potency and continence.”
Average Probability without Timepoint	3 (8%)	“And what I’m telling you is your likelihood of getting back to this level in terms of both continence and potency is about 90%.”
Average Probability with Timepoint	19 (48%)	“I look at [urinary incontinence] as a temporary inconvenience because beyond a year about 10% of men need a pad and the rest are totally continent.”
Precision Estimate	1 (2%)	“In general the curve looks like this: if you look at all men with prostate cancer in both continence and potency is that this is your baseline, 100% of urinary function, continence or potency. This is 3 months, 6, 9, 12. And this is surgery, okay, baseline. Right after surgery things get worse and then they get drastically better between 3 and 6 months. And then they get a little better up to a year and then it falls off. So the statistics we quote out are here… So it’s less of a decremented function and earlier return to baselines. So these are all averages.”
Post-Radiation Erectile Dysfunction		
Not mentioned	15 (42%)	
Name Only	7 (19%)	“All the radiation options are going to carry a risk of erectile dysfunction.”
Generalization	5 (14%)	“Again, your young age and good erectile function would predict you’d do well afterwards.”
Average Percent Incidence without Timepoint	8 (22%)	“But I would say, 40% to 50% of men incur some erectile issue as a result of radiation therapy.”
Average Probability with Timepoint	1 (3%)	“And the way I describe radiation’s effect on the sexual function is usually what it does is it’s sort of like an acceleration of the aging process. So if you took men that are in the 60, 70-year-old age group and you did nothing to them and you followed them for 15 or 20 years, almost every single one of them would lose their function because it’s just over time, you know, that’s sort of the nature of being a human man is the erectile function gets worse when you get into that 80- or 90-year-old range. And so with radiation, the sexual function, that aging process, I would describe it as accelerated, so if it would’ve taken, you know, a certain amount of time, it can, you know, take less time, so if it would’ve taken 15 years, maybe it takes five or six years or seven years to, you know, sort of accelerate to that place. About two thirds of men, if they have a decrease in function from radiation, they can still respond to medicines like Cialis or Viagra or some of these other medicines.”
Precision Estimate		Not observed
Post-radiation lower urinary tract symptoms		
Not mentioned	8 (22%)	
Name Only	17 (47%)	“[Radiation] comes with a little bit of baggage as well, like not incontinence, but it causes, the radiation beam hits the bladder, and it can cause urinary frequency or urgency, waking up at night, those kind of symptoms.”
Generalization	5 (14%)	“And the risks of [urinary symptoms] especially with your pre-existing issues with urination are high. And that makes me worry that we treat you and your life will be made worse by the SEs.”
Average Probability without Timepoint	3 (8%)	“In most men, especially men with good urinary function, the urinary function comes back pretty close to baseline. There’s about 15 percent of men, one in six roughly, that are going to need some type of medication for urinary function that they didn’t need before.”
Average Probability with Timepoint	3 (8%)	“Long term, as we talked about, about 15 or 20 percent of people are going to have just increased irritation of the bladder even after the treatment is done”
Precision Estimate	0 (0%)	N/A

### Radiation SE

Radiation was discussed in 36 of 50 consultations (72%). Coders identified five discrete radiation-related SE discussed in consultations: irritative lower urinary tract symptoms (LUTS), post-radiation ED, bladder and bowel bleeding, secondary malignancy, and bowel dysfunction.

There was wide variation in the most detailed form of communication used at any point during the consultation to describe major radiation SE (Fig. 1, Appendix Table 2). Major radiation-related SE of LUTS, post-radiation ED, bowel/bladder bleeding, secondary malignancy, and bowel dysfunction were not mentioned in 22%(8/36), 42%(15/36), 67%(24/36), 67%(24/36), and 64%(23/36) of consultations, respectively. These same SE were not quantified (mentioned in name only or generalized) in 61%(22/36), 33%(12/36), 22%(8/36), 28%(10/36), and 25%(9/36) of consultations, respectively. They were noted as average incidence without timeline in 8%(3/36), 22%(8/36), 8%(3/36), 0% and 6%(2/36), respectively. In only 3–8% were post-radiotherapy SE quantified with timeline (average probability with timepoint or precision estimate). A wide range of informational quality was again observed across these modes of communication (illustrative quotes in Table 1).

### Variation by physician specialty and tumor risk

**Fig. 2:**
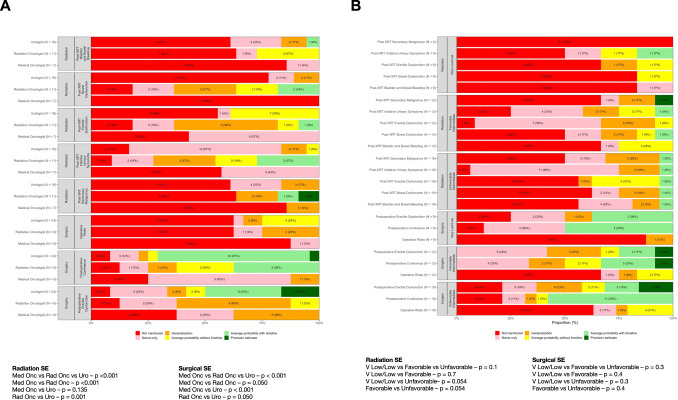
Variation in mode of communication of side effects by treatment type and counseling provider specialty and tumor risk. **A** Variation in Mode of Communication of Side Effects by Treatment Type and Counseling Provider Specialty. Radiation SE. Med Onc vs Rad Onc vs Uro—*p* < 0.001. Med Onc vs Rad Onc—*p* < 0.001. Med Onc vs Uro—*p* = 0.135. Rad Onc vs Uro—*p* = 0.001. Surgical SE. Med Onc vs Rad Onc vs Uro—*p* < 0.001. Med Onc vs Rad Onc—*p* = 0.050. Med Onc vs Uro—*p* < 0.001. Rad Onc vs Uro—*p* = 0.050. **B** Variation in Mode of Communication of Side Effects by Treatment Type and Tumor Risk. Radiation SE. V Low/Low vs Favorable vs Unfavorable—*p* = 0.1. V Low/Low vs Favorable—*p* = 0.7. V Low/Low vs Unfavorable—*p* = 0.054. Favorable vs Unfavorable—*p* = 0.054. Surgical SE. V Low/Low vs Favorable vs Unfavorable—*p* = 0.3. V Low/Low vs Favorable—*p* = 0.4. V Low/Low vs Unfavorable—*p* = 0.3. Favorable vs Unfavorable—*p* = 0.4.

Concordance of physician specialty with SE increased granularity of communication (Fig. 2A), but SE were still frequently not quantified by specialty concordant physicians. When comparing all opportunities to communicate SE, surgeons and radiation oncologists failed to quantify major surgical SE 47%(34/72) and 75%(18/24) of the time (*p* = 0.03), respectively. Conversely, radiation oncologists and surgeons failed to quantify major radiation SE 71%(39/55) and 90%(81/90) of the time(*p* = 0.006). Additionally, medical oncologists never quantified surgical (0/24) or radiation (0/35) SE.

Higher tumor risk did not increase granularity of communication (Fig. 2B). When comparing all opportunities to communicate SE, major surgical SE were not quantified 71%(17/24), 67%(24/36), and 58%(33/57) of the time for very low/low-, favorable-, and unfavorable intermediate-risk disease, respectively (*p* = 0.5). When comparing all opportunities to communicate SE, major radiotherapy SE were not quantified 87%(26/30), 83%(50/60), and 88%(70/80) of the time for very low/low-, favorable-, and unfavorable intermediate-risk disease, respectively (*p* = 0.8).

## Discussion

Effective communication of SE is critical for informed SDM for men with clinically localized prostate cancer. In order for patients to be full partners in SDM, they need to be informed of the risks of therapy to weigh against possible benefits in mitigating cancer progression and mortality. Indeed, information communicated by the physician has been found to be the most important factor affecting patient satisfaction with treatment decisions [9]. The challenge for the counseling physician is how to effectively articulate these risks in a meaningful, informative, and concise manner.

In this study, we found substantial variation in informational quality in communication of major treatment-related SE. At the most superficial level, we found that major SE of radical prostatectomy—ED and UI—were not mentioned in 15% and 12% of consultations, and major SE of radiotherapy—irritative urinary symptoms (LUTS), ED, bowel/bladder bleeding, secondary malignancy, and bowel dysfunction—were not mentioned in 25%, 46%, 61%, 64%, and 75% of consultations, respectively. Moreover, when these risks were articulated, there was wide variation in the amount of detail provided, ranging from no quantification (name of the SE only or a generalization as “high”/”low”) to various forms of numeric quantification (average probability without timeline (i.e., 40% risk of ED), average probability with timeline (i.e., 40% risk of ED at 1 year) or precision estimate based on individual demographics). Overall, surgical risks were quantified in only ~30% of consultations and radiation risks were quantified in only ~20% of consultations. Though the level of detail improved with specialty concordance of SE, significant variation in informational quality persisted even for specialty-concordant risks. Worse tumor risk did not increase level of detail of SE communication. These data argue for better standardization of risk communication so that patients can be adequately informed of the risks of treatment and can participate as true partners with physicians in SDM.

We created a novel, empirically derived framework for how risks of SE are typically communicated in practice. First, the name and a description of the SE are provided. This step is not trivial, as previous work has found that patients do not understand even very basic terminology related to common SE [5]. Next, a variety of methods for attributing likelihood of the event happening are used, ranging from a generalization (“high”/”low”) to a quantification of risk, most often a probability of the outcome. Finally, a timeline for the risk is attributed (e.g., “40% risk of ED at 1 year”). While some SE are assumed to be time-limited and can generally be expressed without a timeline (i.e., operative risks), most prostate cancer treatment SE either improve over time (e.g., ED), remain stable with time (e.g., radiation cystitis/bleeding), or increase with time (e.g., secondary malignancy). Omission of a timeline for risk would imply that the risk is permanent (i.e., “a 30% risk of irritative urinary symptoms after radiation therapy”).

**Fig. 3:**
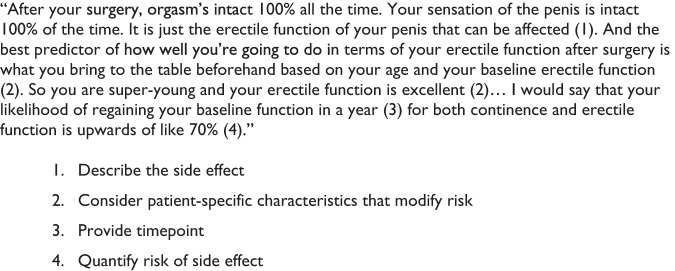
Example of ideal communication strategy for common side effects.

While there is no ideal method for communicating risks of SE, we propose some common-sense recommendations for best practices. First, all major SE should at least be mentioned and briefly described. This allows patients to gauge the basic risks of each treatment option, even if risks are being described to illustrate the major conceptual disadvantages of upfront therapy (i.e., a low-risk patient considering active surveillance). Second, SE should be quantified, especially if the patient is actively considering a particular treatment option (i.e., an unfavorable intermediate-risk patient considering radiotherapy). In our study, this most often took the form of a probability of occurrence. This provides an obvious advantage over a generalization of risk, wherein a physician might describe risk as “high” or “low,” since these terms are relative and depend on how an individual values a particular outcome (i.e., a 10% risk of permanent UI requiring a pad may be “high” or “low” depending on perspective). Third, a timeline for risk should be reported, so patients can gauge whether the risk they are assuming with a particular treatment is permanent or temporary. While it may seem daunting to be able to accomplish these ideals in practice, we provide a real-life example that accomplishes all three ideals in a concise, comprehensive manner (Fig. 3).

Risks of treatment-related SE also differ by patient-specific (e.g., age, comorbidity) [10, 11], treatment-specific (e.g., fractionation of radiation) [12], and physician-specific (e.g., experience) [13] factors. While a multitude of nomograms integrating these factors exist, nomograms are not frequently used since they are cumbersome to use in practice [14]. Electronic medical record-based automated solutions for incorporation of personalized data into decision support pathways will ultimately overcome the technical limitations of incorporating nomogram-based estimates into practice [15]. In the meantime, while it would be convenient to avoid quantification of risk since estimates are flawed, we believe that providing population averages for SE—and noting how these may vary due to patient characteristics and surgeon experience—offers a reasonable estimate for the patient to consider when weighing risks and benefits of treatment. To assist clinicians, we provide a summary of population averages over time for major SE according to major prospective cohort trials (Table 2) [11, 16–22].

**Table 2:** Summary of probability and timeline of common treatment-related side effects from large prospective studies.

SE	Baseline	6 mos	1 year	2 years	3 years	5 years	9 years	15 years
Surgical								
Erectile Dysfunction (insufficient for penetration)								
PCOS	16%^a^	80%^a^	72%^a^	60%^a^	-	77%^b^	-	87%^a^
CAESAR	39%^c^	80%^c^	74%^c^	-	70%^c^	-	-	-
QOL	17%^d^	84%^d^	75%^d^	64%^d^	-	-	-	-
Urinary Incontinence (moderate or big problem in EPIC)								
PCOS	3%^a^	25%^a^	14%^a^	8%^d^	-	14%^b^	-	17%^e^
CAESAR	7%^c^	19%^c^	15%^c^	-	14%^c^	-	-	-
QOL	2%^d^	9%^d^	8%^d^	8%^d^	-	-	-	-
Urinary Incontinence (requiring pads)								
PCOS	2%^a^	50%^a^	27%^a^	21%^a^	-	29%^b^	-	38%^e^
CAESAR	-	-	-	-	-	-	-	-
QOL	1%^d^	34%^d^	24%^d^	20%^d^			-	
Radiation								
Irritative Lower Urinary Tract Symptoms^**f**^								
PCOS	14%^g^	13%^g^	8%^g^	8%^g^	-	9%^b^	-	-
CAESAR	22%^c^	18%^c^	15%^c^	-	15%^c^	-	-	-
QOL	16%^d^	19%^d^	13%^d^	14%^d^	-	-	-	-
Erectile Dysfunction (insufficient for penetration)								
PCOS	42%^g^	54%^g^	54%^g^	63%^g^	-	73%^b^	-	94%^e^
CAESAR	56%^c^	71%^c^	72%^c^	-	71%^c^	-	-	-
QOL	48%^d^	62%^d^	64%^d^	66%^d^	-	-	-	-
Bowel Dysfunction (moderate or big problem in EPIC)								
PCOS	7%^g^	14%^g^	10%^g^	9%^g^	-	5%^b^	-	16%^e^
CAESAR	4%^c^	8%^c^	8%^c^	-	6%^c^	-	-	-
QOL	3%^d^	9%^d^	9%^d^	11%^d^	-	-	-	-
Bowel Bleeding								
PCOS	-	-	-	-	-	-	-	-
CAESAR	1%^c^	1%^c^	2%^c^	-	2%^c^	-	-	-
QOL	1%^d^	1%^d^	5%^d^	5%^d^	-	-	-	-
Secondary Malignancy (RT vs Non-RT)								
Secondary Bladder malignancy	-	-	-	-			1.8% vs 1.1%^h^	
Secondary Colorectal malignancy	-	-	-	-			0.4% vs. 0.3%^h^	

- Indicates not reported.

*PCOS* prostate cancer outcomes study, *CEASAR* comparative effectiveness analysis study of surgery and radiation for clinically localized prostate cancer, *QOL* quality of life.

^a^Stanford et al., JAMA 2000.

^b^Potosky et al., JNCI 2004.

^c^Barocas et al., JAMA 2017.

^d^Sanda et al., NEJM 2008.

^e^Resnick et al.,NEJM 2013.

^f^Irritative lower urinary tract symptoms were reported as frequent urination >1/2 the time in PCOS, and as a moderate or big problem in EPIC in CAESAR and QOL.

^g^Hamilton et al., JCO 2001.

^h^Bagshaw et al., JAMA NO 2022.

There are some limitations that should be considered when interpreting our findings. First, the reported frequencies for SE communication may not represent population averages since our data were limited to a single tertiary referral center. However, measuring quality of communication among fellowship-trained, board-certified physicians who knew they were being recorded would tend to minimize rather than exaggerate our observed findings. Second, the number of consultations within some tumor risk subgroups was low, so quantitative comparisons of these groups may be underpowered to detect smaller differences. Third, numerous factors including health numeracy and literacy may affect how physicians communicate risk; our observations are based on our patient sample that is largely well educated.

## Conclusions

Physician communication of prostate cancer treatment-related SE varies widely in terms of informational quality. We created a framework for how SE are currently communicated and made common sense recommendations for how SE should be ideally communicated, emphasizing disclosure and description of all major SE for treatments under consideration, providing numeric quantification and a timeline for the risk. Our work argues for greater standardization and monitoring of the SE data communicated in consultations so that patients can be sufficiently informed of potential risks. Indeed, we feel that adequate communication of these risks—mentioning all major SE and ideally quantifying them—should be audited as a marker of quality of care. Ensuring adequate patient education about risks (and rewards) of therapy is the only conceivable way that patients can engage as fully informed partners in SDM.

## Supplementary information

Appendix Table 1: Sample Characteristics

Appendix Table 2. Variation in Mode of Communication of Side Effects by Treatment Type

Appendix Table 3. Range of Numeric Estimates Communicated for Side Effects (When Reported)

## Data Availability

Requests for data will be evaluated by the study team on a case-by-case basis.
