# Supplementary material for: Variation in communication of side effects in prostate cancer treatment consultations
Source: Prostate Cancer Prostatic Dis. 2024 Feb 23;28(1):145–52. doi: 10.1038/s41391-024-00806-2 (PMC11341774; doi:10.1038/s41391-024-00806-2)
Supplement: Supplementary file 1 — Appendix Table 1: Sample Characteristics [file 41391_2024_806_MOESM1_ESM.docx]

**Appendix Table 1: Sample Characteristics**

|  | *N=50* |
| --- | --- |
| Age* | 67.0 [61.2;72.8] |
| Race |  |
| Black or African American | 9 (18.0%) |
| White | 41 (82.0%) |
| Stage |  |
| T1a | 1 (2.00%) |
| T1c | 43 (86.0%) |
| T2a | 3 (6.00%) |
| T2b | 1 (2.00%) |
| T2c | 2 (4.00%) |
| PSA* | 6.00 [5.21;10.5] |
| Biopsy Gleason Score |  |
| 3+3 | 11 (22.0%) |
| 3+4 | 24 (48.0%) |
| 4+3 | 15 (30.0%) |
| Prostate Cancer Comorbidity Index Score |  |
| 0 | 13 (30.2%) |
| 1–2 | 16 (37.2%) |
| 3–4 | 5 (11.6%) |
| 5–6 | 4 (9.30%) |
| 7–9 | 4 (9.30%) |
| 10+ | 1 (2.33%) |

* [Median, IQR]
