# Supplementary material for: Variation in communication of side effects in prostate cancer treatment consultations
Source: Prostate Cancer Prostatic Dis. 2024 Feb 23;28(1):145–52. doi: 10.1038/s41391-024-00806-2 (PMC11341774; doi:10.1038/s41391-024-00806-2)
Supplement: Supplementary file 2 — Appendix Table 2. Variation in Mode of Communication of Side Effects by Treatment Type [file 41391_2024_806_MOESM2_ESM.docx]

**Appendix Table 2. Variation in Mode of Communication of Side Effects by Treatment Type**

| **Treatment Type/Side Effect** | **Frequency of Mode of Communication (n, %)** | | | | | | |
| --- | --- | --- | --- | --- | --- | --- | --- |
|  | Not Mentioned | Name Only | Generalization | Average Probability without Timeline | Average Probability with Timeline | Precision Estimate |  |
| **Radical Prostatectomy** | | | | | | |  |
| Postoperative Erectile Dysfunction | 6/40 (15%) | 10/40 (25%) | 9/40 (22%) | 3/40 (8%) | 8/40 (20%) | 4/40 (10%) |  |
| Postoperative Continence | 5/40 (12%) | 9/40 (22%) | 3/40 (8%) | 3/40 (8%) | 19/40 (48%) | 1/40 (2%) |  |
| Operative Risks | 27/40 (68%) | 3/40 (8%) | 4/40 (10%) | 6/40 (15%) | 0/40 (0%) | 0/40 (0%) |  |
| **Radiation Therapy** | | | | | | |  |
| Post-XRT Erectile Dysfunction | 15/36 (42%) | 7/36 (19%) | 5/36 (14%) | 8/36 (22%) | 1/36 (3%) | 0/36 (0%) |  |
| Post-XRT Irritative Urinary Symptoms | 8/36 (22%) | 17/36 (47%) | 5/36 (14%) | 3/36 (8%) | 3/36 (8%) | 0/36 (0%) |  |
| Post-XRT Bowel Dysfunction | 23/36 (64%) | 4/36 (11%) | 5/36 (14%) | 2/36 (6%) | 2/36 (6%) | 0/36 (0%) |  |
| Post-XRT Bladder and Bowel Bleeding | 24/36 (67%) | 6/36 (17%) | 2/36 (6%) | 3/36 (8%) | 1/36 (3%) | 0/36 (0%) |  |
| Post-XRT Secondary Malignancy | 24/36 (67%) | 4/36 (11%) | 6/36 (17%) | 0/36 (0%) | 1/36 (3%) | 1/36 (3%) |  |
