# Supplementary material for: Variation in communication of side effects in prostate cancer treatment consultations
Source: Prostate Cancer Prostatic Dis. 2024 Feb 23;28(1):145–52. doi: 10.1038/s41391-024-00806-2 (PMC11341774; doi:10.1038/s41391-024-00806-2)
Supplement: Supplementary file 3 — Appendix Table 3. Range of Numeric Estimates Communicated for Side Effects (When Reported) [file 41391_2024_806_MOESM3_ESM.docx]

**Appendix Table 3. Range of Numeric Estimates Communicated for Side Effects (When Reported)**

| **Side Effect** | **Range of Numeric Estimates When Reported** |
| --- | --- |
| Postoperative Erectile Dysfunction | 50-90% recover function at one year; 10-20% have permanent erectile dysfunction; 20% never recover function after 2 years; 10-20% never recover function after 2.5 years; 50% recover function (without timeline); 30-50% have erectile dysfunction (without timeline) |
| Postoperative Urinary Incontinence | <10-10% need a pad and <5-5% need surgical intervention after 1 year postop; <10% need a pad after 1 year postop; 5-10% have leakage beyond 18 months; 20-25% wear at least one pad per day (without timeline); 5-10% need surgery to correct leakage (without timeline); 5% will have minimal continence (without timeline) |
| Operative Risks | <1-<10% (without timeline) |
| Post-XRT Irritative Urinary Symptoms | 15% have symptoms at 3-6 months post-XRT and 83% ultimately return to baseline function; 15-20% after treatment is done; 15-20% have symptoms (without timeline) |
| Post-XRT Erectile Dysfunction | 66% will require meds for erectile dysfunction eventually; 20-50% (without timeline) |
| Post-XRT Bladder and Bowel Bleeding | <10% at ten years post-XRT; 1-5% (without timeline) |
| Post-XRT Bowel Dysfunction | 1-2% long-term; 1-2% (without timeline) |
| Post-XRT Secondary Malignancy | <1% over 10-20 years; 1% at 20-25 years |
